# Both Cerebral and Hematopoietic Deficiencies in CCR2 Result in Uncontrolled Herpes Simplex Virus Infection of the Central Nervous System in Mice

**DOI:** 10.1371/journal.pone.0168034

**Published:** 2016-12-08

**Authors:** Rafik Menasria, Coraline Canivet, Jocelyne Piret, Jean Gosselin, Guy Boivin

**Affiliations:** 1 Research Center in Infectious Diseases, CHU of Quebec Research Center, Department of microbiology-immunology and infectious diseases, Faculty of Medicine, Laval University, Quebec City, QC, Canada; 2 Laboratory of Innate Immunity, CHU of Quebec Research Center, Department of Molecular Medicine, Faculty of Medicine, Laval University, Quebec City, QC, Canada; Rutgers University, UNITED STATES

## Abstract

CCR2 is a chemokine receptor expressed on the surface of blood leukocytes, particularly «Ly6C^hi^» inflammatory monocytes and microglia. Signaling through this receptor is thought to influence the immune activity of microglia as well as monocytes egress from the bone marrow (BM) and their trafficking into the central nervous system (CNS) in several neurological diseases. During experimental herpes simplex virus 1 (HSV-1) encephalitis (HSE), CCR2 deficiency has been reported to exacerbate the outcome of the disease. However, the precise contribution of CCR2 expressed in cells of the CNS or peripheral monocytes in the protection against HSE remains unclear. To dissect the differential role of CCR2 during HSE, chimeric mice with receptor deficiency in the brain or blood cells were generated by transplanting wild-type (WT) C57BL/6 or CCR2^-/-^ BM-derived cells in CCR2^-/-^ (WT→CCR2^-/-^) and WT (CCR2^-/-^→WT) mice, respectively. Our results indicate that following intranasal infection with 1.2x10^6^ plaque forming units of HSV-1, CCR2 deficiency in hematopoietic cells and, to a lesser extent, in CNS exacerbates the outcome of HSE. Mortality rates of CCR2^-/-^ (71.4%) and CCR2^-/-^→WT (57.1%) mice were significantly higher than that of WT (15.3%; *P*<0.01 and *P*<0.05, respectively) but the difference did not reach statistical significance for WT→CCR2^-/-^ animals (42.8%; *P* = 0.16). Both peripheral and CNS deficiencies in CCR2 resulted in increased infectious viral titers and wider dissemination of HSV antigens in the brain as well as an overproduction of inflammatory cytokines and chemokines including IL-1β, IL-6, CCL2, CCL3 and CCL5. Furthermore, CCR2 deficiency in the hematopoietic system altered monocytes egress from the BM and their recruitment to the CNS, which may contribute to the failure in HSV-1 containment. Collectively, these data suggest that CCR2 expressed on cells of CNS and especially on peripheral monocytes is important for the control of HSV-1 replication and inflammatory environment during experimental HSE.

## Introduction

Herpes simplex virus encephalitis (HSE) is the most common cause of sporadic viral encephalitis in the Western world, accounting for up to 20% of all cases [1, 2]. HSE affects 2 to 4 individuals per million people per year and can result from either primary or recurrent infection mainly caused by herpes simplex virus 1 (HSV-1) [3–5]. HSE is known to induce severe neuro-inflammation associated with impairment of neurological functions leading to clinical signs such as altered consciousness, abnormal behavior and localized neurological findings including seizure and paralysis [6, 7]. Prior to the introduction of the antiviral drug acyclovir, the mortality rate in HSE patients was over 70%, which has markedly decreased to 10–30% with specific treatment. However, between 25% and 90% of survivors have been reported to develop neurological sequelae [1, 8]. Our understanding of the pathogenesis of HSV-1 infection of the central nervous system (CNS) remains incomplete. It has been suggested that both direct virally-induced and indirect inflammatory-mediated damages are implicated in HSE and death following acute infection [9–11].

The control of HSV-1 infection in the CNS relies on cellular immune responses mediated by local tissue macrophages, namely microglia [12]. It has been reported that microglia are implicated in the control of virus replication in the brain by producing inflammatory cytokines and chemokines such as type I interferons (IFN-I), interleukin (IL)-6, IL-1β, C-X-C motif ligand 10 (CXCL10), C-C motif ligand 2 (CCL2) and CCL5 [12–15]. In addition to microglia, it has been demonstrated that following HSV infection, a high expression of chemokines induced the infiltration of blood monocytes into the CNS through rolling and adhesion mechanisms along the brain endothelium wall [16, 17]. In mice, blood monocytes can be classified into two subsets based on the expression levels of chemokine receptors C-X3-C motif receptor 1 (CX3CR1) and chemokine receptor 2 (CCR2): the inflammatory monocytes (CCR2^+^/CX3CR1^low^/Ly6C^hi^) that are actively recruited into inflamed tissues and contribute to inflammatory responses, and the patrolling monocytes (CCR2^−^/CX3CR1^high^/Ly6C^low^) that exert a surveillance role in the lumen of blood vessels and promote tissue repair [18–25]. Previous results from our laboratory have demonstrated that both monocyte subsets are able to infiltrate the CNS during experimental HSE and may play an important role in elaborating the immune response to HSV-1 infection [26].

Mechanisms controlling monocyte dynamics in blood and their recruitment into inflamed tissues are very complex and require a panel of chemokine receptors and adhesion molecules [19]. Among them, the CCR2 signaling pathway is the most studied with respect to monocytes trafficking. It consists of a G protein-coupled receptor with seven transmembrane domains that can be activated by several chemokines such as CCL7, CCL8, CCL12 and CCL13 although its most potent ligand is CCL2 [27]. Under several pathophysiological conditions, it has been reported that monocytes are mobilized from the bone marrow (BM) to the blood circulation mainly in a CCR2-dependent manner [28–31]. The CCR2 signaling pathway has also been shown to modulate the infiltration of monocytes into inflamed tissues of several organs including the brain, where these cells can play a beneficial or a pathologic role depending on the type of lesions [27, 30, 32–36].

In addition to blood monocytes, CCR2 is thought to be expressed by cells of the CNS. Information regarding the expression of CCR2 at the surface of non-hematopoietic cells in the CNS and its role in immune response are currently limited and vary depending on mouse models of cerebral insults [27]. It is reported that CCR2 is expressed by neurons, astrocytes and particularly microglia in various regions of human brain [27]. Furthermore, it has been shown that CCL2 activates microglia by triggering the release of IL-1β and TNF-α as well as by up-regulating their CCR2 expression level in a mouse model of stroke [37–39]. In contrast, another study using CCR2-red fluorescent protein (RFP) knock-in mice did not confirm that CCR2 was expressed in the CNS. Indeed, no CCR2-RFP signal could be detected in neurons, microglia or astrocytes of mice with experimental autoimmune encephalitis (EAE) [40].

By using CCR2^-/-^ mice, we have previously demonstrated that the deficiency in this signaling pathway results in decreased mobilization of monocytes from the BM to the blood in experimental HSE [33]. Such reduced levels of monocytes in the blood were associated with higher mortality rates in CCR2^-/-^ mice compared to wild-type (WT) C57BL/6 controls [33]. However, this study did not permit to delineate the relative contribution of CCR2 signaling in blood monocytes and cells of the CNS. To circumvent this caveat and better evaluate the impact of CCR2 signaling on the outcome of HSE, we generated chimeric mice lacking this receptor either in cells of the CNS or of the hematopoietic system (i.e., circulating leukocytes). By using these chimeric mouse models, we evaluated the effect of CCR2 deficiency in each of these compartments on the outcome of HSE (survival rates), infectious titers and viral distribution, cytokines/chemokines levels in the brain, and monocytes trafficking. We report here that the CCR2 signaling pathway through blood leukocytes and, to a lesser extent, through cells of the CNS influences the control of HSE.

## Material and Methods

### Animals

Wild-type (WT) C57BL/6 male mice were purchased from Charles River Canada (St-Constant, Quebec, Canada) whereas CCR2^−/−^ (B6.129S4-Ccr2tm1Ifc/J) male mice, which are maintained in a C57BL/6 background, were purchased from the Jackson Laboratory (Bar Harbor, ME). Animals were acclimated to standard laboratory conditions (12 h light/dark cycle; on at 7:15 and off at 19:15) and housed 3 to 4 per cage. All animals were used in accordance to the Canadian Council on Animal Care guidelines and the protocol was approved by the Animal Care Ethics Committee of Laval University (protocol no. 2013078).

### Irradiation and bone marrow transplantation

Chimeric mice deficient in CCR2 either in cells of the CNS or in the hematopoietic system were generated by transplantation of WT bone marrow-derived cells in CCR2^-/-^ mice (WT→CCR2^-/-^) or by injection of cells harvested from CCR2^-/-^ mice to WT animals (CCR2^-/-^→WT), respectively. Eight- to nine-week old WT and CCR2^-/-^ recipient mice were housed in autoclaved cages with irradiated mouse chaw. Mice were treated with 0.2 mg trimethoprim and 1 mg sulfamethoxazole (SEPTRA, GlaxoSmithKline, Mississauga, Ontario, Canada) per mL of sterile water *ad libitum* started 1 week before and maintained for 2 weeks after transplantation. WT and CCR2^-/-^ recipient mice were exposed to 10 Gy total body irradiation using a ^60^Co source (Theratron-780; MDS Analytical Technologies, Concord, Ontario, Canada). Bone marrow cells from age- and sex-matched WT and CCR2^-/-^ donor mice were aseptically harvested by flushing the femurs and tibias with Dulbecco’s phosphate-buffered saline (DPBS) containing 1 g/L glucose and 36 mg/L sodium pyruvate and supplemented with 2% fetal bovine serum (FBS; Wisent, St-Bruno, Quebec, Canada). Cells were filtered through a 40-μm cell strainer (BD Biosciences, San Jose, CA), washed three times in FBS-free DPBS (by centrifugation at 300 × g for 5 min) and counted with a hemacytometer. Recovered cells (1.5×10^7^ in 200 μL DPBS) were then injected in the tail vein of recipient mice 24 h following irradiation [33, 41]. To prevent irradiation-induced dehydration, mice received daily injections of 1 mL saline subcutaneously for one week.

### Experimental procedures

For survival curve experiment, 16-week old WT, CCR2^-/-^, CCR2^-/-^→WT and WT→CCR2^-/-^ mice (n = 14 mice per group) were infected intranasally with a viral inoculum consisting in 1.2x10^6^ plaque forming units (PFU) of HSV-1 clinical strain H25 (grown and passaged in Vero cells) in 20 μL minimal essential medium (MEM) as described elsewhere [42]. HSE-related signs, namely, ruffled fur, ocular swelling, shaking movements, swollen forehead, weight loss and mortality, were monitored twice daily for 20 days. Animals were sacrificed when a ≥ 20% weight loss or a combination of two other obvious sickness signs were observed.

For determination of infectious viral titers as well as cytokine and chemokine levels in brain homogenates, 4 to 5 mice per group were deeply anesthetized with an intraperitoneal injection of a mixture of ketamine hydrochloride (Bioniche Animal Health, Belleville, Ontario, Canada) and xylazine (Bimeda, Cambridge, Ontario, Canada) and sacrificed prior to (day 0) and on days 4, 6, 8 and 10 following infection by intracardiac perfusion with cold 0.9% saline. Brains were then harvested and homogenized in phosphate-buffered saline (PBS) containing protease (cOmplete) and phosphatase (PhosSTOP) inhibitor cocktails (Roche Applied Science, Laval, Quebec, Canada). Samples were stored at -20°C until use.

For immunohistochemistry studies, 4 to 5 mice per group were sacrificed prior to (day 0) and on days 4, 6, 8 and 10 following infection by intracardiac perfusion with cold 0.9% saline followed by a 4% paraformaldehyde solution (pH 7.4) at 4°C. Brains were removed, post-fixed in a paraformaldehyde solution for 24 h, and placed in a 15% sucrose solution prepared in 4% paraformaldehyde at 4°C for at least 48 h. Brains were cut in 25-μm coronal sections on dry ice with a microtome (Reichert-Jung, Cambridge Instruments Company, Deerfield, IL). Sections were collected in a cold cryoprotectant solution (0.05 M sodium phosphate buffer at pH 7.3 containing 30% ethylene glycol and 20% glycerol) and stored at −20°C.

### Infectious viral titers measurements

Virus titers were determined in brain homogenates by a standard plaque assay. Briefly, monolayers of African green monkey kidney (Vero) cells were infected with serial dilutions of brain homogenates for 90 min in a 5% CO_2_ humidified incubator at 37°C. The viral suspensions were then removed, and cells were incubated with MEM containing 2% FBS and 0.4% SeaPlaque agarose (Lonza, Rockland, ME) for 72 h. Cells were fixed and stained with crystal violet, and the number of PFU was determined under an inverted microscope. The limit of detection of the plaque assay is 5 PFU per well.

### Immunohistochemistry analyses

Free-floating brain sections were washed 3 times for 15 min in potassium PBS (KPBS; Sigma-Aldrich, St. Louis, MO) and incubated for 30 min in KPBS containing chicken serum (Rockland Immunochemicals, Limerick, PA), 1% BSA and 0.4% Triton X-100 (both from Sigma-Aldrich). Sections were incubated overnight with a primary antibody, i.e., polyclonal rabbit anti-HSV-1/2 (1:750 dilution) (AbD Serotec, Kidlington, United Kingdom), polyclonal rabbit anti-ionized calcium-binding adaptor molecule-1 (Iba1) (brain macrophages/microglia marker; diluted 1:3000) (Wako Chemicals, Richmond, VA) or polyclonal goat anti-CCR2 (1:1000 dilution) (Novus Biologicals, Littleton, CO), diluted in KPBS containing 0.5% BSA and 0.2% Triton X-100 at 4°C. Sections were then rinsed 3 times for 15 min and incubated with a secondary antibody, i.e., Alexa 594-conjugated chicken anti-rabbit or Alexa 488-conjugated chicken anti-goat (both diluted 1:1500) (Invitrogen, Carlsbad, CA) at room temperature. Finally, nuclear staining was performed using DAPI (diluted at 0.0002% for 10 min; Molecular Probes, Eugene, OR). Sections were then rinsed 3 times for 10 min in KPBS before being mounted onto SuperFrost slides (Fisher Scientific, Nepean, Ontario, Canada) and coverslipped with Fluoromount-G (SouthernBiotech, Birmingham, AL). Standard fluorescence microscopy images were captured using a Nikon Eclipse 80i microscope (Nikon, Melville, NY) equipped with a digital camera (QImaging, Surrey, British Columbia, Canada). Regions of the CNS were identified based on the mouse brain atlas (www.http://atlas.brain-map.org).

### Cytokine and chemokine levels measurements

Brain homogenates (350 μL) of mice sacrificed prior to (day 0) and on days 6, 8 and 10 following the infection were centrifuged at 13,000 × *g* for 10 min at 4°C. A volume of 100 μL of the supernatant was used for cytokine and chemokine measurements using the Bio-Rad Bio-Plex mouse cytokine group I plex assay (Bio-Rad Laboratories, Mississauga, Ontario, Canada) according to the manufacturer’s instructions. Results were analyzed with the Bio-Plex system equipped with the Bio-Plex Manager Software v6.0 (Bio-Rad Laboratories).

### Flow cytometry procedures for evaluation of blood monocytes

Prior to (day 0) and on days 4, 6, 8 and 10 following infection, blood samples (~120 μL) were drawn from the facial vein of CCR2^-/-^→WT and WT→CCR2^-/-^ chimeric mice as well as age- and sex-matched WT and CCR2^-/-^ controls (n = 5 mice per group). Samples were quickly collected in EDTA-coated tubes (Starstedt, Montreal, Quebec, Canada) to prevent coagulation. A volume of 35 μL of DPBS without Ca^2+^ and Mg^2+^ (Sigma-Aldrich) was added to 65 μL of blood and incubated for 20 min on ice with purified rat anti-mouse CD16/CD32 antibody diluted 1:100 (clone 2.4G2; BD Biosciences) to block non-specific binding of IgGs to Fc receptors. Samples were washed and resuspended in 100 μL of DPBS after being centrifuged at 300 x g for 10 min. Cell suspensions were then labeled with the following rat anti-mouse antibodies for 40 min at 4°C: PE-Cy5-CD45 (clone 30-F11; BD Biosciences), APC-CD115 (clone AF598; eBioscience, San Diego, CA), PE-Cy7-CD11b (clone M1/70; eBioscience), V450-Ly6C (clone AL21; BD Biosciences) and PE-Ly6G (clone 1A8; BD Pharmingen, San Jose, CA). Red blood cells were lysed with BD Pharm Lyse™ (BD Biosciences) for 30 min at room temperature, and the recovered leukocytes were washed and resuspended in DPBS. Flow cytometry analyses and data acquisition were performed using a BD SORP LSR II and the BD FACSDiva software, respectively.

### Flow cytometry analyses of brain leucocytes

Prior to (day 0) and on days 4, 6, 8 and 10 following infection, WT, CCR2^-/-^, CCR2^-/-^→WT and WT→CCR2^-/-^ mice (5 animals per group) were deeply anesthetized and perfused intracardially with ice-cold DPBS without Ca^2+^ and Mg^2+^. Brains were extracted and immediately homogenized with a plunger in 20 mL of DPBS supplemented with 0.077 mg of Liberase TL (Roche Diagnostics, Mannheim, Germany) and incubated for 1 h at 37°C. Brain homogenates were filtered through a 70-μm cell strainer (BD Biosciences). The cell suspension was centrifuged at 300 x g for 10 min at room temperature. The supernatant was aspirated and cells were gently resuspended in 7 mL of 37% Percoll (GE Healthcare, Uppsala, Sweden). The cell suspension was underlaid beneath 80% Percoll and centrifuged at 600 x g for 40 min with slow acceleration and deceleration rates. The cell ring at the interphase was collected and mixed thoroughly with DPBS containing 2% FBS. Cells were then centrifuged at 300 x g for 10 min and washed twice with DPBS plus 2% FBS. Cells were first incubated on ice for 35 min with purified rat anti–mouse CD16/CD32 (Mouse Fc Block; clone 2.4G2; BD Biosciences). After a washing step, cells were incubated on ice for 40 min with the same pool of secondary antibodies described above except APC-CD115. Labeled cells were then washed and resuspended in DPBS. Flow cytometry analyses and data acquisition were performed using a BD SORP LSR II and the BD FACSDiva software, respectively.

### Statistical analyses

Differences in mouse survival rates were compared using a log-rank (Mantel-Cox) test. Differences in infectious viral titers, cytokine and chemokine levels and percentages of infiltrating cells in brain homogenates were evaluated using a one-way analysis of variance (ANOVA) with Tukey's multiple comparison post-test. All statistical analyses were carried out using the GraphPad Prism software program, version 5.00 (GraphPad Software, San Diego, CA). A *P* value ≤ 0.05 was considered statistically significant.

## Results

### CCR2 is expressed by resident microglia following HSV-1 intranasal infection of mice

In this study, we generated chimeric mice with CCR2 deficiency affecting either resident cells of the CNS or hematopoietic cells by the reconstitution of irradiated CCR2^-/-^ and WT mice with WT (WT→CCR2^-/-^) or CCR2^-/-^ (CCR2^-/-^→WT) bone marrow cells, respectively. Eight weeks post-transplantation, mice were infected intranasally with 1.2x10^6^ PFU of HSV-1. Age- and sex-matched total body CCR2^-/-^ and WT C57BL/6 mice were used as controls. It is well accepted that CCR2 could be expressed by blood leukocytes including monocytes but its expression by resident microglia of the CNS may vary depending on mouse models of diseases [18, 27]. We have thus evaluated the expression of this receptor in brain macrophages prior to and following infection with HSV-1 by immunohistochemistry analyses in all groups of mice. No immuno-reactivity for CCR2 could be detected prior to infection in all groups of mice (data not shown). Interestingly, CCR2-positive cells that also express the microglia marker Iba1 were detected in brain sections of the olfactory bulb (not shown) and the medulla of WT, CCR2^-/-^→WT and WT→CCR2^-/-^ mice on days 6 (S1 Fig) and 8 (not shown) post-infection (p.i.) whereas no signal was detected in CCR2^-/-^ group. We cannot exclude that CCR2-positive cells detected in brains of WT and WT→CCR2^-/-^ animals could be blood leucocytes, which are known to infiltrate the CNS following infection with HSV-1 in our mouse model of HSE [26]. However, in the CCR2^-/-^→WT group, CCR2 expression is restricted to resident cells of the CNS since infiltrating cells are CCR2-negative.

### CCR2 deficiency in the hematopoietic system results in higher mortality rates during HSE

**Fig 1:**
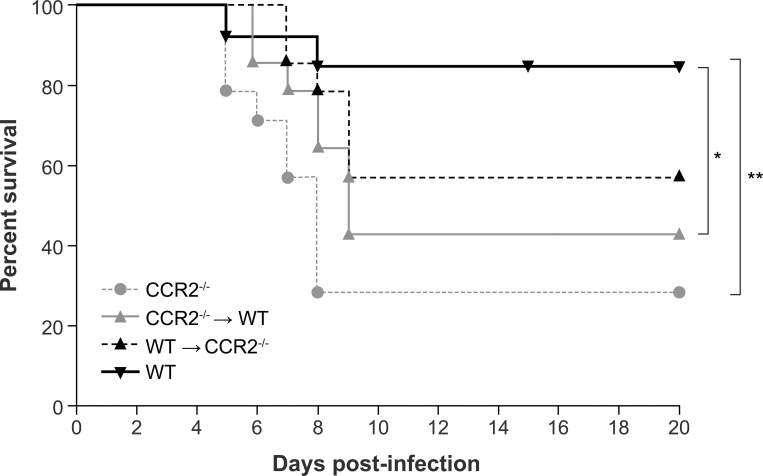
Effect of CCR2 deficiency in resident cells of the CNS or in the hematopoietic system on mouse survival rates during HSE. Eight-week old recipient wild-type (WT) C57BL/6 and CCR2^-/-^ mice conditioned with irradiation were transplanted with bone marrow cells harvested from CCR2^-/-^ (CCR2^-/-^→WT) and WT (WT→CCR2^-/-^) animals, respectively. Total body CCR2^-/-^ and WT mice were used as controls. Sixteen-week old WT, CCR2^-/-^, CCR2^-/-^→WT and WT→CCR2^-/-^ mice (n = 14 mice per group) were infected intranasally with HSV-1 (1.2x10^6^ plaque forming units (PFU) in 20 μl minimal essential medium). Mice were examined carefully twice a day for 20 days, and two obvious signs of sickness or a ≥ 20 % weight loss were considered as endpoints for sacrifice. Survival curves were analysed using a log-rank (Mantel-Cox) test. Statistically significant results between groups are indicated as follows: *, *P*<0.05; **, *P*<0.01.

To evaluate the relative impact of a deficiency in the CCR2 signaling pathway during HSE, WT, CCR2^-/-^→WT, WT→CCR2^-/-^ and CCR2^-/-^ mice were infected intranasally with 1.2x10^6^ PFU of HSV-1 and were examined daily for up to 20 days p.i. Mortality rates of CCR2^-/-^ (71.4%) and CCR2^-/-^→WT (57.1%) mice were significantly higher than that of WT (15.3%; *P*<0.01 and *P*<0.05, respectively) (Fig 1). CCR2 deficiency in resident cells of the CNS (WT→CCR2^-/-^) also increased susceptibility to the infection but the mortality rate was not statistically different compared to WT (42.8%; *P* = 0.16). Taken together, these data suggest that complete CCR2 deficiency and that localized in the hematopoietic system significantly increases the mortality rates in our mouse model of HSE.

### CCR2 deficiency in resident cells of the CNS or in the hematopoietic system is associated with higher viral replication in the CNS during HSE

**Fig 2:**
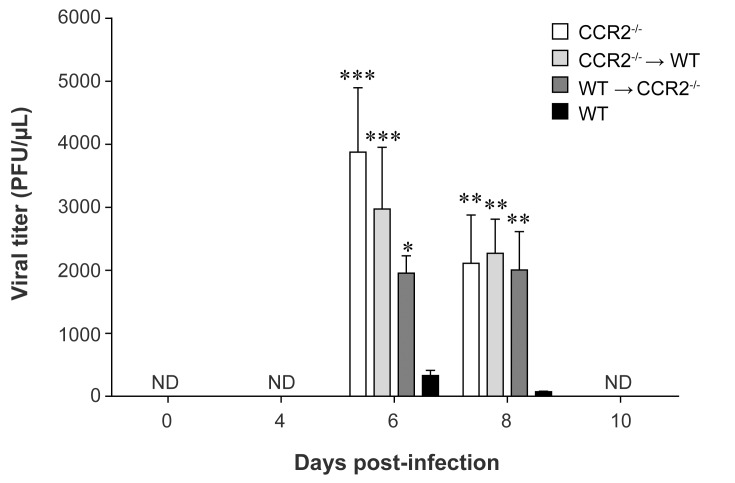
Impact of CCR2 deficiency in resident cells of the CNS or in the hematopoietic system on the control of viral replication during HSE. Wild-type (WT) C57BL/6, CCR2^-/-^, CCR2^-/-^→WT and WT→CCR2^-/-^ mice were sacrificed prior to (day 0) and on days 4, 6, 8 and 10 following intranasal infection with 1.2x10^6^ PFU of HSV-1. Viral titers were measured in brain homogenates by a standard plaque assay on Vero cells. Results are reported as PFU/μL of brain homogenates and represent the means ± standard deviations of 4 to 5 mice per group at each time point. Statistical analyses were performed using a one-way analysis of variance (ANOVA) with Tukey’s multiple comparison post-test. Statistically significant results compared to those for the WT group are indicated as follows: *, *P*<0.05; **, *P*<0.01; ***, *P*<0.001. ND, not detected.

Infectious viral titers were measured in brain homogenates of all groups of mice prior to (day 0) and on days 4, 6, 8 and 10 following intranasal infection with HSV-1. These different time points were selected based on survival curves (Fig 1). As shown in Fig 2, no virus was detectable on day 0 and on day 4 p.i. Virus titers were then significantly increased on day 6 in CCR2^-/-^ (*P*<0.001), CCR2^-/-^→WT (*P*<0.001) and WT→CCR2^-/-^ (*P*<0.05) mice as well as on day 8 p.i (*P*<0.01 for the three groups) compared to WT. These data suggest that CCR2 deficiency throughout the body, in the hematopoietic system or in resident cells of the CNS, results in loss of HSV-1 containment.

### CCR2 deficiency in resident cells of the CNS or in the hematopoietic system influences HSV-1 dissemination in the brain

**Fig 3:**
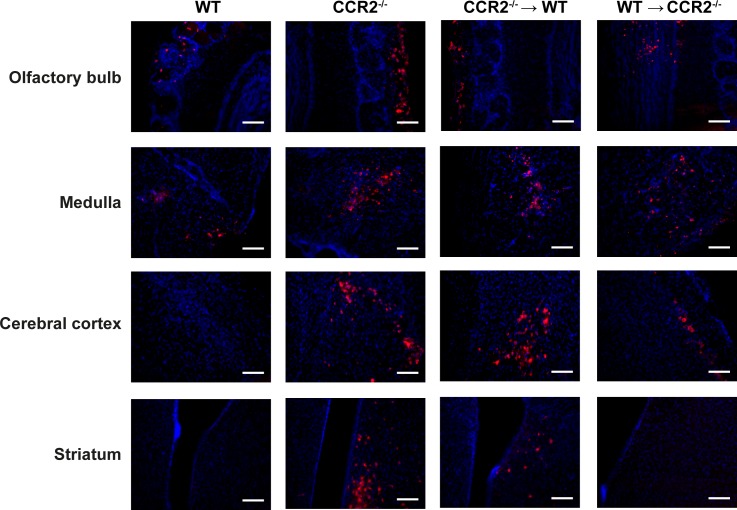
Impact of CCR2 deficiency in resident cells of the CNS or in the hematopoietic system on HSV-1 distribution in the brain during HSE. Representative micrographs illustrating the distribution pattern of HSV-1 proteins in the olfactory bulb, the medulla, the cerebral cortex and the striatum of wild-type (WT) C57BL/6, CCR2^-/-^→WT, WT→CCR2^-/-^ and CCR2^-/-^ animals (4 to 5 mice per group) sacrificed on day 6 post-infection. Brain slices (25 μm thick) were processed for immunohistochemistry analyses with a polyclonal rabbit anti-HSV-1/2 primary antibody and an Alexa 594-conjugated chicken anti-rabbit secondary antibody (red), followed by nuclear staining with DAPI (blue). Scale bar 100 μm.

The distribution of HSV-1 antigens was evaluated by immunohistochemistry analyses in the brain of WT, CCR2^-/-^, CCR2^-/-^→WT and WT→CCR2^-/-^ groups following intranasal infection. In WT animals sacrificed on days 6 (Fig 3) and 8 (data not shown) following the infection, HSV-1 antigens could be found in the olfactory bulb, the hypothalamic lateral ventricle (data not shown) and the medulla whereas they were no longer visible in these regions by day 10 following infection (data not shown). These results are in line with previous studies conducted in our laboratory [26]. Interestingly, in addition to the anatomical areas described above, the distribution of HSV-1 proteins was extended to the cerebral cortex of CCR2^-/-^, CCR2^-/-^→WT and WT→CCR2^-/-^ mice on day 6 p.i. In CCR2^-/-^ and CCR2^-/-^→WT groups, HSV-1 proteins could also be detected in the striatum. These data suggest that the loss of CCR2 signaling in hematopoietic cells and, to a lesser extent, in the CNS may result in a wider HSV-1 dissemination in the brain in our mouse model of HSE.

### CCR2 deficiency in resident cells of the CNS or in the hematopoietic system results in an increased pro-inflammatory environment in the brain

**Fig 4:**
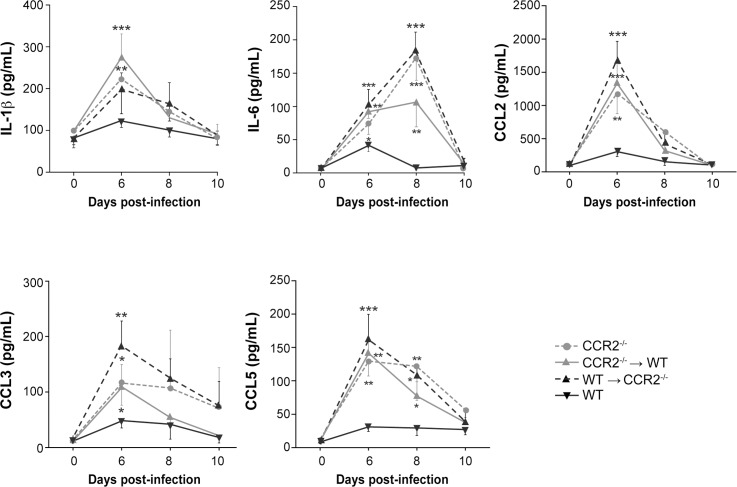
Impact of CCR2 deficiency in resident cells of the CNS or in the hematopoietic system on cytokines and chemokines production during HSE. Levels of cytokines and chemokines in brain homogenates of wild-type (WT) C57BL/6, CCR2^-/-^, CCR2^-/-^→WT and WT→CCR2^-/-^ mice were evaluated prior to (day 0) and on days 6, 8 and 10 following intranasal infection with 1.2x10^6^ PFU of HSV-1. Levels of cytokines and chemokines were measured using magnetic bead-based immunoassay. Results are expressed in pg/mL of brain homogenates and represent the means ± standard deviations of 4 to 5 mice per group at each time point. Statistical analyses were performed using a one-way analysis of variance (ANOVA) with Tukey’s multiple comparison post-test. Statistically significant results obtained in CCR2^-/-^, CCR2^-/-^→WT and WT→CCR2^-/-^ compared to those of the WT group are indicated as follows: *, *P*<0.05; **, *P*<0.01; ***, *P*<0.001.

In order to evaluate whether the immune response could contribute to the higher mortality rates observed in CCR2^-/-^, CCR2^-/-^→WT and WT→CCR2^-/-^ groups, cytokine and chemokine levels in the brain of chimeric and deficient mice were compared to those of WT mice. Levels of IL-1β, IL-6, CCL2, CCL3 and CCL5, which are known to play an important role during HSE, were measured in brain homogenates using magnetic bead-based immunoassays prior to (day 0) and on days 6, 8 and 10 following infection. In the WT group, Fig 4 shows that levels of IL-1β, IL-6, CCL2 and CCL3 significantly increased on day 6 p.i. compared to their counterparts sacrificed on day 0 (*P*<0.05, *P*<0.001, *P*<0.01 and *P*<0.05, respectively (not indicated)). Levels decreased thereafter on days 8 and 10 p.i. to reach baseline. The production of IL-1β also increased following the infection in CCR2^-/-^, CCR2^-/-^→WT and WT→CCR2^-/-^ (*P*<0.05 on day 6 p.i. (not indicated)) compared to their respective counterparts sacrificed on day 0 and then decreased on day 8 to reach baseline level on day 10. Importantly, levels of IL-1β were significantly higher than those of WT on day 6 p.i. in the more susceptible CCR2^-/-^ (*P*<0.01) and CCR2^-/-^→WT mice (*P*<0.001). In addition, levels of IL-6, CCL2, CCL3 and CCL5 in CCR2^-/-^, CCR2^-/-^→WT and WT→CCR2^-/-^ mice also increased following the infection (*P*<0.05 (not indicated)) compared to their counterparts sacrificed on day 0 and were significantly higher than those of WT mice on day 6 (*P*<0.05 for all deficient groups). Levels of IL-6 and CCL5 remained significantly increased in these mice on day 8 (*P*<0.05) compared to the WT group but not those of CCL2 and CCL3. On day 10 p.i., levels of all cytokines/chemokines in CCR2^-/-^, CCR2^-/-^→WT and WT→CCR2^-/-^ animals were comparable to those of WT mice. Overall, these data suggest that CCR2 deficiency in cells of the CNS and in the hematopoietic system results in an increase production of cytokines and chemokines, which could amplify the inflammatory immune response and exacerbate the disease.

### CCR2 deficiency in the hematopoietic system alters monocyte levels in the blood

**Fig 5:**
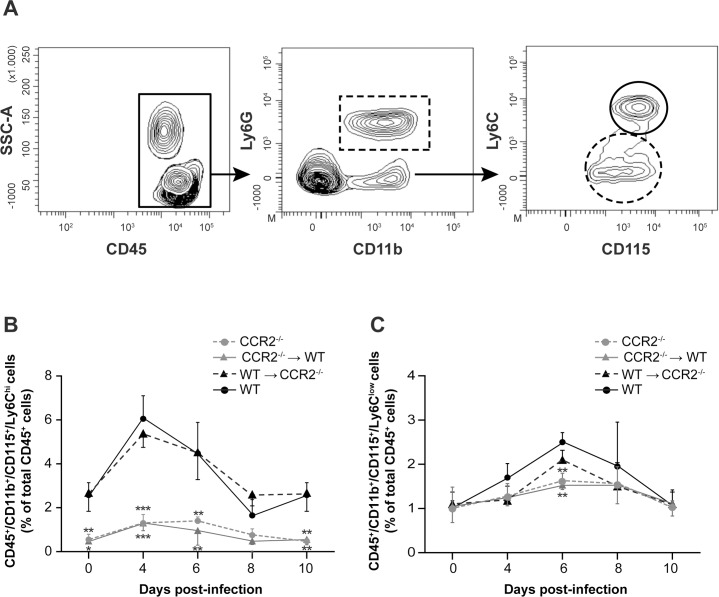
Impact of CCR2 deficiency in resident cells of the CNS or in the hematopoietic system on blood monocytes mobilization during HSE. Blood leukocytes of wild-type (WT) C57BL/6, CCR2^-/-^, CCR2^-/-^→WT and WT→CCR2^-/-^ mice were analysed by flow cytometry prior to (day 0) and on days 4, 6, 8 and 10 following intranasal infection with 1.2x10^6^ PFU of HSV-1. (A) Representative flow cytometry contour plots showing the gating strategy for blood leukocytes (i.e., CD45^+^) (continuous rectangle), granulocytes (CD45^+^/CD11b^+^/Ly6G^+^) (dashed rectangle), inflammatory monocytes (CD45^+^/CD11b^+^/CD115^+^/Ly6C^hi^) (continuous circle) and patrolling monocytes (CD45^+^/CD11b^+^/CD115^+^/Ly6C^low^) (dashed circle). (B) Histograms illustrating the percentage of inflammatory monocytes compared to total leukocytes (CD45^+^), (C) histograms showing the percentage of patrolling monocytes compared to total leukocytes. Statistical analyses were performed using a one-way analysis of variance (ANOVA) with Tukey's multiple comparison post-test. Statistically significant results compared to those of the WT group are indicated as follows: *, *P*<0.05; **, *P*<0.01; ***, *P*<0.001.

Levels of blood leukocytes in CCR2^-/-^, CCR2^-/-^→WT, WT→CCR2^-/-^ and WT mice were evaluated by flow cytometry analyses prior to (day 0) and on days 4, 6, 8 and 10 following infection with HSV-1. In our study, the expression of the cell surface marker CD45 was first detected to target blood leukocytes (Fig 5A). Monocytes were differentiated according to CD115 and Ly6C expression levels into «Ly6C^hi^» and «Ly6C^low^» cells which were associated with inflammatory (CD45^+^/CD11b^+^/CD115^+^/Ly6C^hi^) and patrolling (CD45^+^/CD11b^+^/CD115^+^/Ly6C^low^) monocytes, respectively.

In the WT group, 2.5%±0.5 of all blood leukocytes (i.e., CD45^+^) expressed the CD115 and «Ly6C^hi^» markers prior to infection and were attributed to inflammatory monocytes (Fig 5B). This percentage significantly increased on day 4 compared to day 0 (6.5%±1.0; *P*<0.001 (not indicated)) and then decreased on day 6 (4.4%±1.4) to finally reach the baseline on days 8 (1.6%±0.7) and 10 (2.4%±0.6) following infection. A similar kinetics pattern was observed in WT→CCR2^-/-^ animals. Interestingly, levels of inflammatory monocytes also increased significantly on day 4 p.i. in CCR2^-/-^ and CCR2^-/-^→WT groups (1.4%±0.3 and 1.3%±0.4 (*P*<0.05, both not indicated), respectively) compared to their respective counterparts sacrificed on day 0 (0.6%±0.2 and 0.5%±0.1, respectively). Levels decreased on day 8 to reach baseline on day 10 following infection. However, inflammatory monocytes in the blood in these two groups were significantly lower than those of WT and WT→CCR2^-/-^ groups prior to as well as on days 4 and 6 p.i. (P<0.05). These data suggest that CCR2 signaling pathway in hematopoietic cells is important for the egress of inflammatory monocytes from the bone marrow to the blood.

Fig 5C shows that in the WT group, levels of «Ly6C^low^» patrolling monocytes increased from 1.0%±0.1 on day 0 to 1.7%±0.3 on day 4 and reached a peak level on day 6 p.i. (2.5%±0.2; *P*<0.01 (not indicated)). Levels then decreased on day 8 (1.9%±1.0) to reach baseline on day 10 p.i. A similar kinetics was also observed in WT→CCR2^-/-^, CCR2^-/-^→WT and CCR2^-/-^ mice. However, on day 6 p.i., levels of patrolling monocytes in CCR2^-/-^→WT and CCR2^-/-^ groups were significantly lower compared to WT (*P*<0.01 for both groups). Overall, these results indicate that CCR2 signaling in cells of the hematopoietic system is involved in the mobilization of both monocyte subsets into the blood following infection in our mouse model of HSE.

### CCR2 deficiency in resident cells of the CNS or the hematopoietic system results in altered monocytes infiltration into the brain

**Fig 6:**
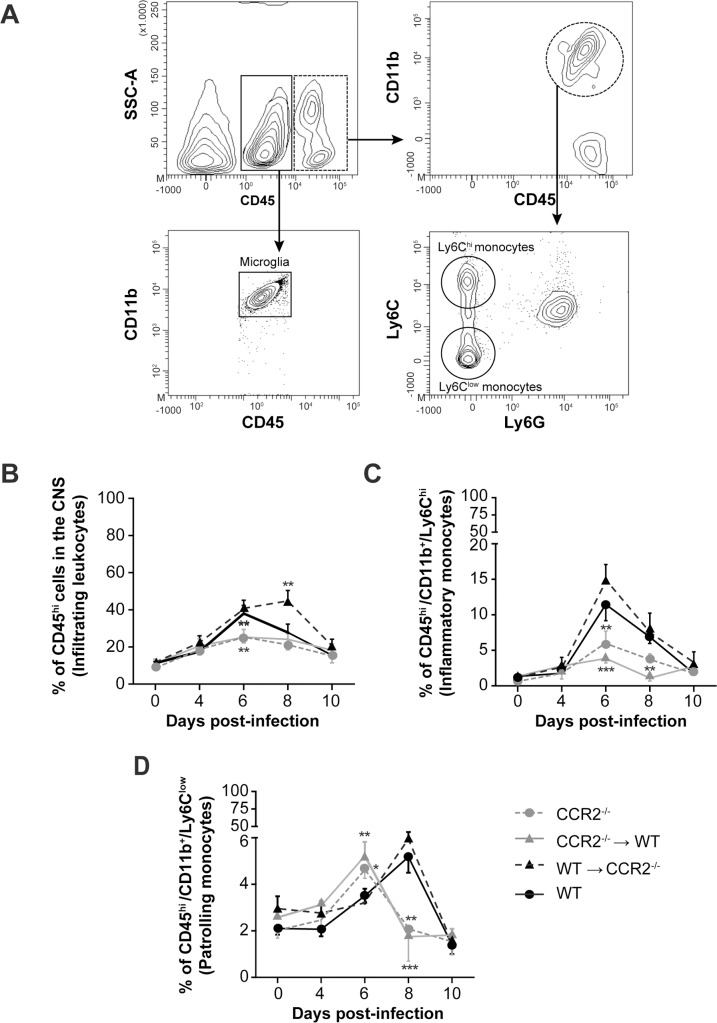
Effects of CCR2 deficiency in resident cells of the CNS or in the hematopoietic system on blood leukocytes infiltration into the CNS during HSE. Leukocytes of wild-type (WT) C57BL/6, CCR2^-/-^, CCR2^-/-^→WT and WT→CCR2^-/-^ mice were analysed in brain homogenates by flow cytometry prior to (day 0) and on days 4, 6, 8 and 10 following intranasal infection with 1.2x10^6^ PFU of HSV-1. **(A)** Flow cytometry contour plots illustrating the gating strategy used for brain leukocytes differentiation in mouse sacrificed on day 6 following infection. The CD45 marker was used to discriminate microglia (i.e., CD45^low^; continuous rectangle) from infiltrating leukocytes (i.e., CD45^hi^; dashed rectangle). Among «CD45^hi^/CD11b^+^» infiltrating myeloid cells (dashed circle), monocytes subsets were discriminated according to Ly6C expression level into «Ly6C^hi^» inflammatory and «Ly6C^low^» patrolling monocytes. The percentages of **(B)** infiltrating leukocytes, **(C)** inflammatory monocytes and **(D)** patrolling monocytes with respect to total brain leukocytes represent the means ± standard deviations of 4 to 5 mice per group at each time point. Statistical analyses were performed using a one-way analysis of variance (ANOVA) with Tukey’s multiple comparison post-test. Statistically significant results compared to those of WT animals are indicated as follows: *, *P*<0.05; **, *P*<0.01; ***, *P*<0.001.

The infiltration of blood leukocytes into the CNS of CCR2^-/-^, CCR2^-/-^→WT, WT→CCR2^-/-^ and WT mice was evaluated by flow cytometry analyses prior to (day 0) and on days 4, 6, 8 and 10 following infection with HSV-1. In our study, the expression levels of CD45 and CD11b were used to distinguish «CD45^low^/CD11b^+^» from «CD45^hi^/CD11b^+^» cells which were attributed to resident microglia and infiltrating leukocytes, respectively (Fig 6A). Infiltrating monocytes were further differentiated according to Ly6C expression level into «Ly6C^hi^» inflammatory and «Ly6C^low^» patrolling monocytes, respectively.

Fig 6B shows that in the WT group, 11.3%±0.9 of all brain leukocytes (i.e., CD45^+^) expressed the «CD45^hi^» marker on day 0 and were attributed to infiltrating immune cells. This percentage significantly increased on days 6 (38.0%±4.3; *P*<0.001 (not indicated)) and 8 (28.2%±4.5; *P*<0.001 (not indicated)) and then almost returned to the baseline on day 10 (16.7%±4.2) p.i. In WT→CCR2^-/-^ mice, the infiltration kinetic of blood leukocytes was almost similar to that of WT on days 4 and 6 but continued to increase on day 8 (44.9%±6.2; *P*<0.001 (not indicated)) and then decreased on day 10 p.i. (25.5%±7.5; *P*<0.05 (not indicated)) compared to their counterparts sacrificed on day 0. This increase was also significant compared to WT group on day 8 p.i. (*P*<0.01, Fig 6B). Levels of «CD45^hi^» cells were increased on day 6 p.i. in CCR2^-/-^ group (from 8.9%±1.4 on day 0 to 24.5%±4.6; *P*<0.001 (not indicated)) and CCR2^-/-^→WT mice (from 12.6%±1.6 to 31.8%±8.3; *P*<0.001 (not indicated)). Levels decreased on day 8 for both CCR2^-/-^ (21.5%±2.0; *P*<0.001 (not indicated)) and CCR2^-/-^→WT (24.1%±3.4; *P*<0.001 (not indicated)) to reach baseline levels on day 10 p.i. Importantly, these levels were significantly lower than that of WT group on day 6 (*P*<0.01 for CCR2^-/-^ and *P*<0.01 for CCR2^-/-^→WT) and then decreased to similar levels as WT on days 8 and 10 p.i. Overall, our results indicate that the lack of CCR2 signaling pathway throughout the body and more specifically in the hematopoietic compartment alters the infiltration of blood leukocytes into the CNS during HSE.

Fig 6C shows that levels of «Ly6C^hi^» inflammatory monocytes in WT mice were slightly increased on day 4 (from 1.3%±0.1 on day 0 to 1.7%±0.4) and were significantly higher on days 6 (11.5%±2.2; *P*<0.001 (not indicated)) and 8 (6.8%±0.9; *P*<0.01 (not indicated)) following infection. Their amount then decreased on day 10 p.i. to reach baseline level (1.7%±0.2). The infiltration kinetic of «Ly6C^hi^» monocytes in WT→CCR2^-/-^ mice was similar to that of WT but levels were slightly higher than WT on day 6 without reaching statistical significance. Levels of «Ly6C^hi^» monocytes also increased on day 6 p.i. in CCR2^-/-^ (from 1.0%±0.1 prior to infection to 5.9%±1.7; *P*<0.001 (not indicated)) and CCR2^-/-^→WT (from 1.3%±0.5 to 3.8%±1.0; *P*<0.05 (not indicated)). Their amounts then decreased on days 8 and 10 following infection to reach baseline levels. Importantly, the amounts of inflammatory monocytes were significantly lower in CCR2^-/-^ (*P*<0.01) and CCR2^-/-^→WT (*P*<0.001) animals on day 6 and in CCR2^-/-^→WT (*P*<0.01) on day 8 p.i. than those of WT group. Globally, these data suggest that a complete CCR2 deficiency, and particularly that in the hematopoietic compartment, results in a lack of inflammatory monocytes infiltration, which could alter the immune response to HSV-1 infection.

Fig 6D shows that in WT mice, «Ly6C^low^» patrolling monocytes invaded the CNS on day 6 p.i. (from 2.3%±0.2 on day 0 to 3.5%±0.3; *P*<0.05 (not indicated)) and reached a peak level on day 8 (5.1%±0.7; *P*<0.001 (not indicated)) to finally decrease on day 10 (1.7%±0.5) p.i. This infiltration pattern is in line with previous studies from our laboratory showing the delayed infiltration of this monocyte subset during HSE [26]. Earlier (day 6) and higher levels of patrolling monocytes were observed in the CNS of CCR2^-/-^ (4.7%±0.4; *P*<0.05) and CCR2^-/-^→WT (5.2%±0.5; *P*<0.01) mice compared to WT. On day 8 following infection, levels decreased significantly in these groups *(P*<0.01 for CCR2^-/-^ and *P*<0.001 for CCR2^-/-^→WT) compared to WT mice to reach baseline amounts on day 10. Levels of patrolling monocytes in WT→CCR2^-/-^ mice were slightly higher than those of WT on day 8 p.i. but the difference did not reach statistical significance. These data suggest that patrolling monocytes trafficking appears to be influenced by CCR2 deficiency localized in the hematopoietic system.

## Discussion

Monocytes recruitment into the CNS is considered a common feature of viral encephalitis. It is believed that monocytes infiltration into the CNS is critical for pathogens clearance and recovery from infection. However, infiltrating monocytes may also cause brain tissue damages and contribute to more severe symptoms [43]. The CCR2/CCL2 axis is pivotal for leukocyte trafficking, particularly monocytes, in the context of viral encephalitis [35]. Previous work from our laboratory using all body CCR2-deficient mice has shown that the lack of this receptor worsens the outcome of HSE [33]. To further investigate and to delineate the impact of CCR2 during HSE, we generated BM chimeric mice differentially lacking CCR2 in resident cells of the CNS (microglia) or in the hematopoietic system.

Our results confirm that CCR2 plays an important role in protecting against HSE since all body deficiency in this receptor resulted in markedly decreased survival rates. In line with our results, it has been demonstrated that CCR2 signaling pathway is critical for monocytes mobilization in the blood and survival in a mouse model of West Nile virus (WNV) encephalitis [44]. In addition, the lack of CCR2 results in increased mortality and impaired leukocytes activation following infection of the CNS with a neurotropic coronavirus [45, 46]. In a model of Alzheimer’s disease, CCR2^-/-^ mice have been found to exhibit impaired microglial recruitment in injured areas, which results in increased mortality [35, 36, 47, 48].

Furthermore, our data suggest that CCR2 deficiency in the hematopoietic system or in resident cells of the CNS may influence the control of HSV-1 infection with a significant impact on mortality when CCR2 signaling is lacking in the periphery. Indeed, our results showed that infectious viral titers were increased in the brain of CCR2-deficient mice compared to resistant WT animals regardless of the compartment lacking this receptor (CNS *vs* hematopoietic system). These data suggest that CCR2 signaling in resident cells of the CNS may be involved in viral containment mechanisms since reconstitution of CCR2^-/-^ recipient mice with WT hematopoietic cells was not sufficient to control the infection. In this context, the role played by CCR2 signaling in the modulation of the cerebral immune response is not completely elucidated. It has been demonstrated that microglia express CCR2 upon stimuli or lesions and that this receptor plays a role in the immunological response of these cells as well as in their recruitment to injured brain areas [49–51]. Furthermore, CCR2 deficiency also resulted in altered microglial activation following LPS challenge [38].

Hematopoietic deficiency in CCR2 also resulted in higher viral titers and wider distribution of HSV-1 antigens in the brain, which were associated with a marked decrease in the recruitment of blood inflammatory monocytes. Thus, we suggest that the inefficient viral containment in CCR2^-/-^ and CCR2^-/-^→WT mice may be related to the altered recruitment of inflammatory monocytes in the brain. Indeed, it is believed that the CCR2/CCL2 axis is a potent pathway for «Ly6C^hi^» inflammatory monocytes recruitment into the CNS in models of stroke [52], Alzheimer’s disease [47, 48] and EAE [22, 40]. In the context of viral encephalitis, alteration or blockade of the CCR2/CCL2 axis can significantly reduce the infiltration of inflammatory monocytes into the infected brain [15, 21, 33, 53]. In addition, «Ly6C^hi^» inflammatory monocytes appear to play an important role in viral containment. Indeed, higher mortality rates and increased brain viral loads were reported in HSV-2 [54], coronavirus [55] and mouse hepatitis virus (MHV) [46] infections when these cells are depleted. Our hypothesis is corroborated by a study using a mouse model of WNV infection which demonstrated that CCR2 deficiency results in higher brain viral load which was associated with a reduction of «Ly6C^hi^» monocytes infiltration in the CNS, supporting a protective role for these cells [44].

Recent studies have identified a function for CCR2 in the mobilization of monocytes from the bone marrow to the blood [34, 56, 57]. It was demonstrated that CCR2^−/−^ mice are severely monocytopenic and this is dependent on both CCL2 and CCL7 [58]. Similar to our results, monocytopenia was observed in blood of CCR2^-/-^ mice infected with WNV [44]. However, this study suggested that CCR2 deficiency alters inflammatory monocytes accumulation in the blood but did not affect their recruitment into the CNS. As expected, our results showed that CCR2 deficiency in the hematopoietic system resulted in an important blood monocytopenia. Indeed, levels of inflammatory monocytes were decreased prior to and following infection in the blood of CCR2^-/-^ and CCR2^-/-^→WT mice. Thus, the lack of inflammatory monocytes recruitment observed in the CNS of mice having a peripheral deficiency may either be due to the monocytopenia or the absence of CCR2-dependent monocytes trafficking from the blood to the brain. On the other hand, levels of patrolling monocytes, for which recruitment was not shown to be related to CCR2 signaling, only decreased on day 6 p.i. in the blood of mice with peripheral deficiency compared to WT group but these cells were able to infiltrate the CNS despite their low levels in the blood. However, patrolling monocytes were recruited earlier in these mice indicating that CCR2 deficiency may influence their kinetics of infiltration.

Overall, our data suggest that mice carrying a hematopoietic deficiency in CCR2 may not be able to overcome monocytopenia during HSV-1 infection and that the lack of this receptor affects particularly the recruitment of inflammatory monocytes, which appears to be needed for the control of HSE.

It is known that HSV-1 infection of the CNS induces cytokines and chemokines production that play an important role in the control of HSE. Indeed, the control of HSV-1 infection requires the expression of a series of cytokines, mainly IFN-I, IL-1β and IL-6 [14, 59]. Other studies have also shown the involvement of chemokines since the expression levels of CCL2, CCL3, CCL5, CXCL9 and CXCL10 increased in the brain of mice infected with HSV-1 [16, 17]. However, an excessive production of these immune mediators could be associated with an exacerbation of the disease and increased lethality [9, 11]. In this respect, the increased mortality rates observed in CCR2-deficient mice and when deficiency affects hematopoietic cells, were associated with elevated inflammatory cytokines (IL-1β and IL-6) and chemokines (CCL2, CCL3 and CCL5) production in the brain compared to WT. Indeed, it has been already reported that chemokine receptors deficiency may result in increased inflammatory cytokines and chemokines production during CNS insults, which could increase brain damages as well as lethality [16, 17, 44, 60]. Furthermore, the excessive production of chemokines, such as CCL2, CCL3 and CCL5, observed in the more susceptible groups could induce the recruitment of peripheral immune cells such as natural killer (NK) cells and T cells that in turn may contribute to amplify the global inflammation triggered by the virally-infected cells [11]. It will be of interest to further investigate the dynamics of these leukocytes and to address the impact of CCR2 deficiency on their recruitment to the CNS during HSE.

One limitation of our study is that the myeloablative process consisting in total body irradiation can result in minimal non-specific cell engraftment in the CNS. However, this method was shown to be very effective to allow a stable and very high chimerism in the blood and bone marrow of transplanted mice compared to other techniques such as parabiosis, head-shielded irradiation and chemotherapy [41].

In conclusion, our data demonstrate that CCR2 could exert a critical protective role in a mouse model of HSV-1 encephalitis. Our results show for the first time that this chemokine receptor may have an impact on viral containment and inflammatory environment when expressed in resident cells of the CNS and especially in cells of the hematopoietic system.

## Supporting Information

S1 Fig Microglia express CCR2 in the CNS following HSV-1 infection. WT, CCR2^-/-^→WT, WT→CCR2^-/-^ and CCR2^-/-^ mice were infected with HSV-1 via the intranasal route and sacrificed prior to and on days 4, 6, 8 and 10 following infection. The expression of CCR2 by brain macrophages was assessed in brain sections by double immunostaining with goat anti-CCR2 and rabbit anti-Iba1 followed by secondary antibodies, Alexa 488-conjugated chicken anti-goat (green) and Alexa 594-conjugated chicken anti-rabbit (red), respectively. Nuclear staining with DAPI is shown in blue. Pictures depicted here are representative of the region of the medulla of mice sacrificed on day 6 following the infection. White arrows indicate double positive cells. Scale bar 100 μm.
(PDF)
