# Supplementary figures and images for: Both Cerebral and Hematopoietic Deficiencies in CCR2 Result in Uncontrolled Herpes Simplex Virus Infection of the Central Nervous System in Mice
Source: PLoS One. 2016 Dec 8;11(12):e0168034. doi: 10.1371/journal.pone.0168034 (PMC5145225; doi:10.1371/journal.pone.0168034)

**WT**

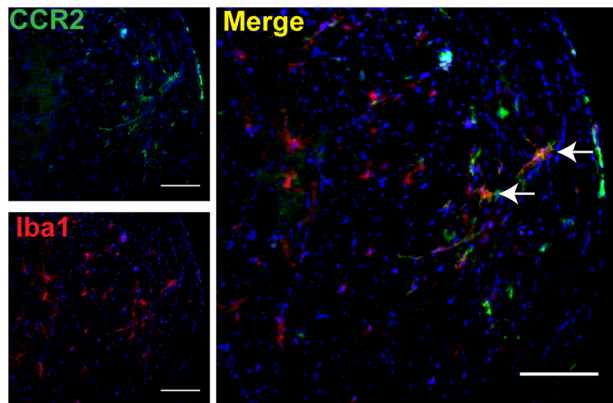

**CCR2<sup>-/-</sup> → WT**

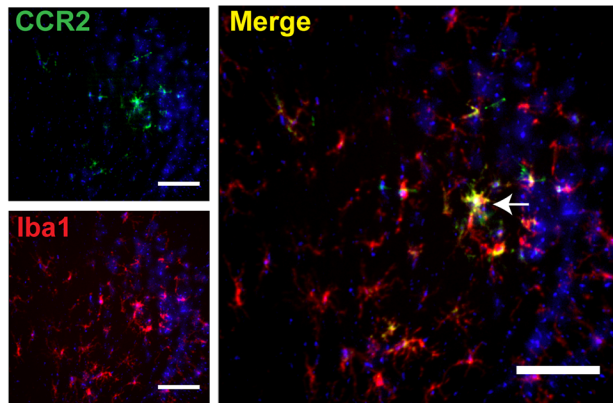

**WT → CCR2<sup>-/-</sup>**

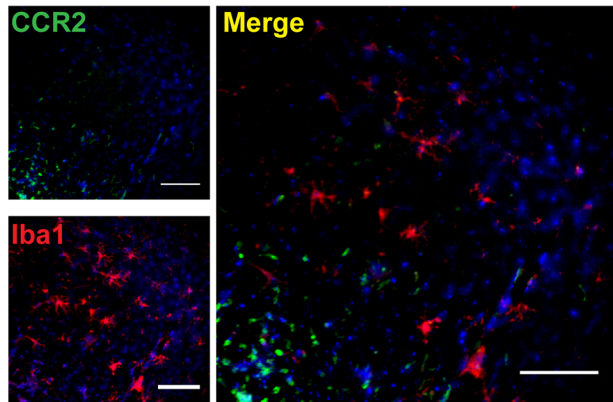

**CCR2<sup>-/-</sup>**

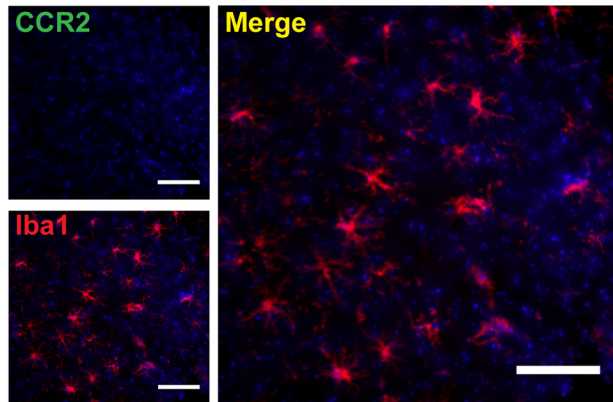

Supplement: S1 Fig — WT, CCR2-/-→WT, WT→CCR2-/- and CCR2-/- mice were infected with HSV-1 via the intranasal route and sacrificed prior to and on days 4, 6, 8 and 10 following infection. The expression of CCR2 by brain macrophages was assessed in brain sections by double immunostaining with goat anti-CCR2 and rabbit anti-Iba1 followed by secondary antibodies, Alexa 488-conjugated chicken anti-goat (green) and Alexa 594-conjugated chicken anti-rabbit (red), respectively. Nuclear staining with DAPI is shown in blue. Pictures depicted here are representative of the region of the medulla of mice sacrificed on day 6 following the infection. White arrows indicate double positive cells. Scale bar 100 μm. (PDF) [file pone.0168034.s001.pdf]
